# Positive Emotion Facilitates Cognitive Flexibility: An fMRI Study

**DOI:** 10.3389/fpsyg.2017.01832

**Published:** 2017-10-31

**Authors:** Yanmei Wang, Jie Chen, Zhenzhu Yue

**Affiliations:** ^1^Faculty of Education, East China Normal University, Shanghai, China; ^2^The School of Psychology and Cognitive Science, East China Normal University, Shanghai, China; ^3^Department of Psychology, Capital Normal University, Beijing, China; ^4^Department of Psychology, Sun Yat-sen University, Guangzhou, China

**Keywords:** positive emotion, dACC, task switching, cognitive flexibility, conflict

## Abstract

Cognitive flexibility is the ability to switch rapidly between multiple goals. By using a task-switching paradigm, the present study investigated how positive emotion affected cognitive flexibility and the underlying neural mechanisms. After viewing pictures of different emotional valence (positive, negative, or neutral), participants discriminated whether a target digit in a specific color was odd or even. After a series of trials, the color of target stimuli was changed, i.e., the switch condition. Switch costs were measured by the increase of reaction times (RTs) in the switch trials compared to those in the repeat trials. Behavior results indicated that switch costs significantly decreased in the positive emotional condition, and increased in the negative emotional condition, compared with those in the neutral condition. Imaging data revealed enhanced activation in the dorsal anterior cingulate cortex (dACC) in switch trials than those in repeat trials. Moreover, the interaction between emotion (positive, negative, neutral) and trial type (repeat vs. switch) was significant. For switch trials, the activation of dACC decreased significantly in the positive condition, while increased significantly in the negative condition compared to neutral condition. By contrast, for repeat trials, no significant difference was observed for the activation of dACC among three emotional conditions. Our results showed that positive emotions could increase the cognitive flexibility and reduce the conflict by decreasing the activation of dACC.

## Introduction

The generation of appropriate action requires the ability to select suitable response among competing possibilities, while inhibiting task-irrelevant responses. These abilities are generally called ‘cognitive flexibility’ (Rende, 2000). Studies of cognitive flexibility have been investigated with diverse cognitive tasks, including task switching (Woodward et al., 2002; Druey, 2014), attention shifting (Wager et al., 2004), and so on. Task switching is a typical cognitive task to investigate cognitive flexibility (Deák and Wiseheart, 2015; Fröber and Dreisbach, 2016). In a task switching task, participants are required to switch between two targets (at least occasionally) or between two tasks (Kiesel et al., 2010). Reaction times (RTs) are significantly longer for switch trials than those for repeat trials, i.e., the so-called “switch cost” (Hübner and Druey, 2008; Kieffaber et al., 2013).

Previous functional magnetic resonance imaging (fMRI) studies consistently reported that anterior cingulate cortex (ACC), which was involved in task switching and cognitive flexibility (Halari et al., 2009; Hyafil et al., 2009; Hakun and Ravizza, 2012), monitored conflict and detected errors (Botvinick et al., 2004; Silvetti et al., 2011). For example, Kim et al. (2014) found that RTs for an incongruent trial were significantly faster when the incongruent trial followed one or several consecutive incongruent trials than when the incongruent trial followed one or several consecutive congruent trials, i.e., the conflict adaptation effect. Conflict adaptation effect was accompanied by reduced activation of ACC and increased activation of dorsal lateral prefrontal cortex (DLPFC).

It has been shown that ACC not only monitors information of conflict and demands, but also responds to many events involving negative emotion, such as monetary loss, pain, and negative feedback etc. (Botvinick, 2007). Although previous evidence shows that ACC is involved in emotion and cognitive flexibility, it remains unclear whether the conflict-related activity of ACC could be modulated by emotional states. It has been well-known that emotion affects a broad range of cognitive processes, e.g., visual processing, working memory, attentional allocation, cognitive control, and social categorization (Foster et al., 2008; Levens and Phelps, 2008; van Steenbergen, 2015). Positive emotion could improve performance in creative problem-solving tasks (Phillips et al., 2002) and insight problems (Subramaniam et al., 2009). In Phillips et al. (2002) study, participants in a specific emotional state were required to retrieve words that met specific criteria in a short period of time. Their results showed that participants in the positive mood reported more words than those in the neutral mood, indicating that positive emotion promoted cognitive flexibility in the word fluency task.

Recent studies showed that positive emotion could enhance cognitive flexibility. A number of studies demonstrated that positive emotion could reduce response conflict and suppress competing response (Van der Stigchel et al., 2011). For example, in a Simon or Flanker task, significant conflict effect was observed in neutral trials, but not in positive trials (Kanske and Kotz, 2011a,b; Xue et al., 2013), indicating that positive emotion reduced response conflict. Nadler et al. (2010) studied the effect of mood states on cognitive processing with a category learning task in which participants were required to classify stimuli by rule-described categories. Music and video clips were adopted to induce happy or sad mood. Their results showed that the performance in the happy mood was better than that in the sad mood. Yang and Yang (2014) adopted a modified Dimensional Change Card Sort (DCCS) task to examine the effect of mood states on the cognitive flexibility. They found that positive emotion improved performance in DCCS task via attenuating switch costs.

Event-related potentials (ERPs) studies also showed that emotion influenced the amplitude of the error-related negativity (ERN), which could be attributed to an incorrect response and might reflect the affective evaluation of errors (Hajcak, 2012). For example, amplitude of ERN reduced under positive affection induced by movie clips (van Wouwe et al., 2011). By contrast, negative affection induced by International Affective Picture System (IAPS) pictures and negative feedback resulted in increase of ERN amplitude (Wiswede et al., 2009).

However, some studies did not find positive emotions promote cognitive flexibility (Bruyneel et al., 2013) or even found that positive emotions impaired cognitive flexibility (Sacharin, 2009). The inconsistency in empirical findings indicated that the association between positive emotions and cognitive flexibility was unclear. For example, Nath and Pradhan (2014) induced positive emotion through positive/neutral writing or watching movie. Participants were required to perform a shape detection task in order to measure cognitive flexibility. Their results showed that RTs between the positive state and the neutral state did not differ significantly. A number of other studies using various tasks of measuring cognitive flexibility, like Stroop task (Phillips et al., 2002), fluency tasks (Carvalho and Ready, 2010), attentional orienting task (Compton et al., 2004), anti-saccade task (Van der Stigchel et al., 2011), did not show conclusive results.

One reason for these inconsistent results might be due to lack of controlling the arousal intensity. According to the motivational dimension model of emotion, high-arousal positive emotion which is high in approach motivation (e.g., desire) narrows cognition, whereas low-arousal positive emotion which is low in approach motivation (e.g., pleasant, happy) broadens attention (Gable and Harmon-Jones, 2010). In the present study, we controlled the arousal level of emotional state and investigated whether positive emotion could improve cognitive flexibility. Moreover, we were interested in the neural correlates of the modulation of emotion on cognitive flexibility. Positive, neutral or negative emotional states of moderate intensity were induced by viewing emotional pictures. Task switching paradigm used by Dreisbach and Goschke (2004) was adopted, and participants were required to judge a digit in a specific color was odd or even while ignoring a digit distractor in another color simultaneously. After several trials, the color of target digit changed to a new color that did not appear before, while distractors were presented in the same color as the former target. We hypothesized that moderate positive emotions of low approach-related motivation could reduce switch cost and facilitate task switching. Since resolving conflict was involved in task switching (Costa and Friedrich, 2012), we hypothesized that the activation of ACC would be larger in the switch condition than that in the repeat condition, which should also be modulated by emotion states.

## Materials and Methods

### Participants

Nineteen undergraduates (6 males, mean age 20.5 years, range 19-21 years) participated in the experiment. They were right-handed, and had normal or corrected-to-normal vision. All participants were free of neurological disorders, psychiatric problems or head trauma and were not under any medication. Participants provided written informed consent and the study was approved by the Ethics Committee of Department of Psychology, Capital Normal University. At the end of the study, participants were paid for taking part in the study.

### Materials

Eighty-four emotional pictures were selected from IAPS (Lang et al., 1998). For each kind of pictures (positive, negative, or neutral), twenty-eight pictures were selected and all items were mild-arousal pictures. Pleasant pictures included lovely babies, animals or beautiful scenes, etc. Unpleasant pictures included frightening animals, human violence, and garbage, etc. Neutral pictures consisted of inanimate objects, such as chair, cup, book, etc. One hundred and ten undergraduate students rated pleasure and arousal values for all pictures. Valence ratings ranged from 1 (very unhappy) to 9 (very happy) and arousal ratings ranged from 1 (very calm) to 9 (very excited). The mean valence and arousal ratings from IAPS norms for the positive pictures were 7.79 (*SD* 2.38) and 5.60 (*SD* 2.06), for the negative pictures were 2.54 (*SD* 1.14) and 6.00 (*SD* 2.87), and for neutral pictures (control) were 4.50 (*SD* 2.23) and 3.05(*SD* 1.89). Negative pictures and positive pictures differed significantly only in valence [*t*(54) = -14.22, *p* < 0.001], but not in arousal [*t*(54) = 0.98, *p* > 0.05].

### Procedure and Design

**FIGURE 1:**
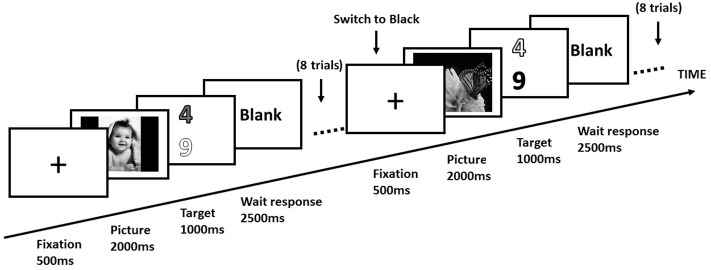
Design of experimental trials in a block. Each participant performed consecutive eight trials in a block. In a switch trial, the target digit was switched to a new color (i.e., black), and the color of distractor (i.e., white) was the same as the previous target.

Participants lay supine in the scanner, viewing the screen through a mirror mounted on the head-coil. Participants engaged in a switch task similar to Dreisbach and Goschke (2004) experimental task (as shown in **Figure 1**). For each trial, a white fixation cross (1° × 1° of visual angle) was presented at the center of the screen. After duration of 500 ms, an emotional picture was presented at the center of the screen for 2000 ms. Then two digits in different colors (red, blue, green, or yellow) were presented simultaneously for 1000 ms. One digit was odd, and the other was even. The target was presented randomly at the upper or lower part of the screen. Participants were instructed to discriminate that digits in a specific color were odd or even. The color of target and distractor was kept constant for eight consecutive trials. After eight consecutive trials, an instructional word indicated a new color of the target (different from the color of earlier targets and distractors) and the color of the distractor was the same as the color of the former target. Each participant performed this task for 3 imaging sessions. Each session consisted of 3 blocks, and 28 trials of the same emotional condition in each block. These 28 different emotional pictures were randomized in each block. The temporal order of 3 blocks was randomized for each participant. In each block, a switch occurred after every eight consecutive trials. The first four trials were excluded from the final analysis. For each switch, we analyzed the consecutive four trials before the switch (*repeat trials*) and the consecutive four trials after the switch (*switch trials*). Switch cost was calculated by subtracting the mean RTs of task-repeat trials (consecutive four trials before a switch) from those of task-switch trials (consecutive four trials after a switch).

Therefore, the present study involved a 3 (Emotion valence: positive, negative vs. neutral) × 2 (Trial type: switch vs. repeat) within-participants design. All participants completed a training session outside the scanner before the scanning.

### fMRI Data Acquisition

All scanning was done with a 1.5 Tesla G.E. SIGNA Scanner (GE Medical Systems), equipped for echo-planar images (EPI). A standard RF head coil was used. Head movement was restricted using a form-fitting vacuum cushion. Functional data were acquired by using a T2^∗^-weighted gradient shot echo-planar imaging sequence with the following parameters: TR = 2000 ms; TE = 40 ms; flip angle = 90°; matrix = 64 × 64; field of view (FOV) = 24 cm × 24 cm. Sixteen transversal slices of 5 mm thickness that covered the whole brain were acquired sequentially with a 1.5 mm gap. High-resolution T1-weighted structural images were also acquired in sagittal planes.

### Functional Image Analysis

Image pre-processing and subsequent analyses were undertaken using statistical parametric mapping (SPM)^1^ on a Matlab platform. High-resolution 1 mm × 1 mm × 1 mm anatomical images were collected for anatomic localization and co-registration. Prior to statistical analysis, head motion was analyzed by center of mass measurements in three orthogonal planes. Data of one participant was excluded from final data analysis due to excessive movement (greater than 3 mm). MNI coordinates were finally converted into Talairach coordinates using the Talairach Daemon database.

At the first level, the general linear model was used to construct a multiple regression design matrix. Six experimental conditions were modeled: (1) neutral_repeat trials (the consecutive four trials before a switch under neutral emotion); (2) neutral_switch trials (the consecutive four trials after a switch under neutral emotion); (3) positive_repeat trials (the consecutive four trials before a switch under positive emotion); (4) positive_switch trials (the consecutive four trials after a switch under positive emotion); (5) negative_repeat trials (the consecutive four trials before a switch under negative emotion); (6) negative_switch trials (the consecutive four trials after a switch under negative emotion). The six head movement parameters derived from the realignment procedure were also included in the first-level GLM and we followed standard processing steps. The regressors for trial types were generated by convolving the boxcar function (representing the presentation time of stimuli of each condition) with the standard HRF provided in SPM. The obtained individual contrast images of the first-level analysis were then entered into a second-level random effects group analysis (one-sample *t*-test) to identify voxels activated during each experimental condition. Statistical threshold of *p* < 0.0001 was used (uncorrected and threshold at 25 voxels). All coordinates given in this article referred to original Talairach space.

Three kinds of comparisons were defined. Firstly, for neural substrates specifically involved in task switch irrespective of emotion, they should be localized by the main effect of trial type, “Switch (positive_switch + neutral_switch + negative_switch) > Repeat (positive_repeat + neutral_repeat + negative_repeat).” Secondly, for neural substrates specifically involved in emotion irrespective of task switching, they should be localized by the main effect of emotion, “Positive (positive_repeat + positive_switch), Neutral (neutral_repeat + neutral_switch) vs. Negative (negative_repeat + negative_switch).” Finally, to directly test our hypothesis, neural correlates which are involved in evaluating the influence of emotion on task switching, should be localized by the neural interaction between emotional conditions and trial type, “Positive (positive_switch vs. positive_repeat) vs. Neutral (neutral_switch vs. neutral_repeat”; “Negative (negative_switch vs. negative_repeat). We used GLM results as a functional mask and then computed the mean parameter estimates over all voxels within the intersection of the functional mask and the anatomical mask for each experimental condition and each participant. These mean parameter estimates were then submitted to a group level ANOVA.

## Results

### Behavioral Results

Trials that involved incorrect responses and RTs exceeding 3 standard deviations from mean RTs (2%) were eliminated from the data. Behavioral data in all conditions were shown in **Table 1**. The switch cost was calculated by subtracting mean RTs of repeat trials (4 trials pre-switch) from mean RTs of switch trials (4 trials post-switch). A 3 (Emotion valence: positive, negative vs. neutral) × 2 (Trial type: switch vs. repeat) analysis of variance (ANOVA) revealed a significant main effect of Trial type, *F*(1,17) = 31.22, *p* < 0.001, $\eta_{p}^{2}$ = 0.34, indicating that RTs were significantly longer in the switch trials (934.00 ms) than those in the repeat trials (881.41 ms). The main effect of Emotion valence was significant, *F*(2,34) = 17.50, *p* < 0.001, $\eta_{p}^{2}$ = 0.27, indicating that RTs were significantly longer in the negative condition (975.29 ms) than those in the neutral condition (885.32 ms) and the positive condition (862.07 ms).

**Table 1:** Mean RTs for each experimental condition.

	Emotion valence		
	
	Neutral	Positive	Negative
Repeat	857.04 (82.21)	852.07 (85.93)	935.12 (99.55)
Switch	913.60 (87.06)	872.95 (76.1)	1015.46 (107.55)


*SD in parenthesis.*

**FIGURE 2:**
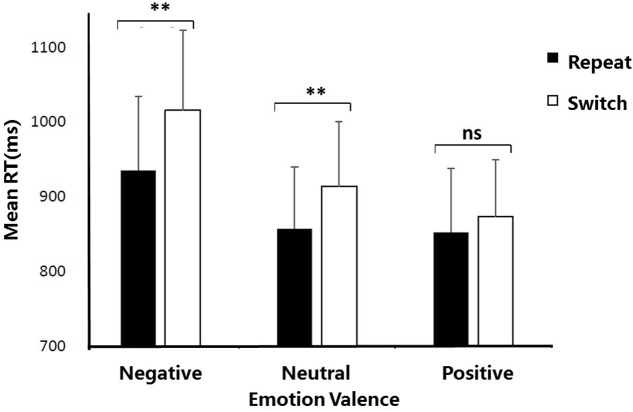
Mean reaction times as a function of Emotion valence and Trial type. ^∗^*p* < 0.05; ^∗∗^*p* < 0.01; ns, no significance. Error bars indicate the standard deviation.

Most importantly, the interaction between Emotion valence and Trial type was significant, *F*(2,34) = 9.98, *p* < 0.01, $\eta_{p}^{2}$ = 0.16. Further planned *t*-tests on simple effects revealed that RTs were significantly longer for switch trials than for repeat trials both in the negative condition (1015.46 ms vs. 935.12 ms), *t*(17) = 3.31, *p* < 0.01, Cohen’s *d* = 1.05, and in the neutral condition (913.60 ms vs. 857.04 ms), *t*(17) = 2.68, *p* < 0.05, Cohen’s *d* = 0.98. By contrast, no significant switch cost (switch vs. repeat trials) was observed in the positive condition, *t*(17) = 0.78, *p* = 0.57, indicating that the positive emotion reduced the switch cost and thereby improving the cognitive flexibility. **Figure 2** illustrates mean RTs as a function of Emotion valence and Trial type.

Reaction times of the first two trials after each switch were further compared with RTs of the next two trials, and the results supported that switch effect decreased gradually after each switch. In positive emotion condition, planned *t*-tests revealed that RTs for the first two trials (893.45 ms) was longer than those for the next two trials (852.45 ms) after each switch, *t*(17) = 2.04, *p* = 0.057. Similarly, there was a trend that RTs for the first two trials (925.71 ms; 1035.85 ms) were longer than RTs for the next two trials (901.49 ms; 995.07 ms) after each switch in both neutral condition [*t*(17) = 1.85, *p* = 0.081] and negative emotion condition [*t*(17) = 1.96, *p* = 0.067].

To observe strong switch effect, we then used the first two trials after each switch as the ‘switch’ condition, since RTs of the first two trials after switch was longer than those of the next two trials. A 3 (Emotion valence: positive, negative vs. neutral) × 2 (Trial type: switch vs. repeat) analyses of variance (ANOVA) revealed a significant main effect of Trial type, *F*(1,17) = 38.25, *p* < 0.001, $\eta_{p}^{2}$ = 0.69, indicating that RTs were significantly longer in the switch trials (951.67 ms) than those in repeat trials (881.41 ms). The main effect of Emotion valence was significant, *F*(2,34) = 19.15, *p* < 0.001. $\eta_{p}^{2}$ = 0.53, indicating that RTs were significantly longer in the negative emotional condition than those in the neutral condition and positive emotional condition. The interaction between Emotion valence and Trial type was significant, *F*(2,34) = 5.57, *p* < 0.01, $\eta_{p}^{2}$ = 0.25. This supplementary analysis using two trials after switch as “switch” condition showed similar ANOVA results as using four trials after switch as “switch” condition. Therefore, we modeled the consecutive four trials after each switch as “switch” condition in the following imaging analysis considering the statistical power.

### Imaging Results

The fMRI BOLD data were analyzed in the following steps: Firstly, we analyzed within-participants mean BOLD signal changes using contrasts specifying the main factors of the Trial type (switch vs. repeat trials) and Emotion valence (positive, negative vs. neutral emotion). This contrast helped to define the brain regions related to task switch and emotional state respectively. Secondly, we investigated the modulation of emotion valence on task switching by analyzing the interaction between Emotion valence (positive, negative, vs. neutral emotion) and Trial type (switch vs. repeat trials).

#### Brain Regions Related to Emotion “Positive/Negative vs. Neutral”

**FIGURE 3:**
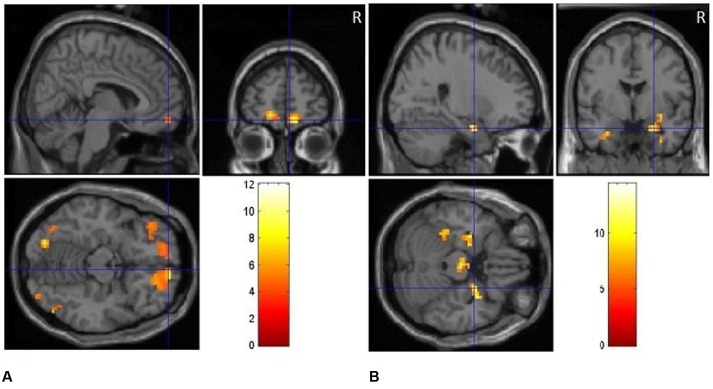
**(A)** Clusters of significant activation for positive picture processing relative to neutral pictures. **(B)** Clusters of significant activation for negative picture processing relative to neutral pictures.

The positive vs. neutral contrast resulted in significantly enhanced BOLD responses in medial [BA10, *t*(1,17) = 4.38, *p* < 0.01] and middle prefrontal cortex [BA11, *t*(1,17) = 3.95, *p* < 0.05], medial orbitofrontal cortex [BA11, *t*(1,17) = 4.29, *p* < 0.01], occipital gyrus [BA18, *t*(1,17) = 4.25, *p* < 0.01; BA19, *t*(1,17) = 4.17, *p* < 0.01], as shown in **Table 2** and **Figure 3A**. The negative vs. neutral contrast revealed significantly enhanced BOLD responses in amygdala, [*t*(17) = 4.33, *p* < 0.01], parahippocampal gyrus [BA 36, BA34, *t*(17) = 3.88, *p* < 0.05], putamen [*t*(17) = 3.96, *p* < 0.05], fusiform gyrus [*t*(17) = 3.65, *p* < 0.05], middle frontal gyrus [BA10, *t*(17) = 4.09, *p* < 0.05], and occipital gyrus [*t*(17) = 3.58, *p* < 0.05], as shown in **Table 2** and **Figure 3B**.

**Table 2:** Anatomical localization of clusters in positive and negative emotional contrasts.

Brain regions	BA	Voxels	Cluster peak (mm)			*t*
**Positive > neutral**						
*Frontal lobes*						
Medial prefrontal cortex	10	136	-12	51	5	4.38
Superior frontal gyrus	10	136	-27	53	-3	4.05
Middle frontal gyrus	11	136	-28	49	-11	3.95
Medial OFC	11	77	6	55	-13	4.29
Superior frontal gyrus	10	77	21	57	-3	3.76
*Occipital lobes*						
Inferior occipital gyrus	18	226	33	-86	-7	4.46
R middle occipital gyrus	18	226	36	-87	2	4.25
L inferior occipital gyrus	18	104	-48	80	1	4.23
L middle occipital gyrus	19	75	-27	-90	-15	4.17
*Subcortical*						
Limbic lobe Uncus	28	35	-27	-90	-15	4.24
**Negative > neutral**						
*Frontal lobes*						
L middle frontal gyrus	10	30	-30	50	60	4.09
*Occipital lobes*						
R middle occipital gyrus	19	30	-30	-80	20	3.58
*Subcortical*						
R amygdala		60	21	-7	17	4.33
L parahippocampal gyrus	34	48	-30	-7	-22	3.88
L fusiform gyrus	33	40	-36	-39	-16	3.65
L putamen	33	35	-18	3	0	3.96


*The coordinates (x, y, z) are in the Talairach coordinates. BA, Brodmann’s areas; L, left; R, right. Displayed are the coordinates of the maximally activated voxel within a significant cluster.*

#### Brain Regions Related to Task Switching “Switch vs. Repeat”

**FIGURE 4:**
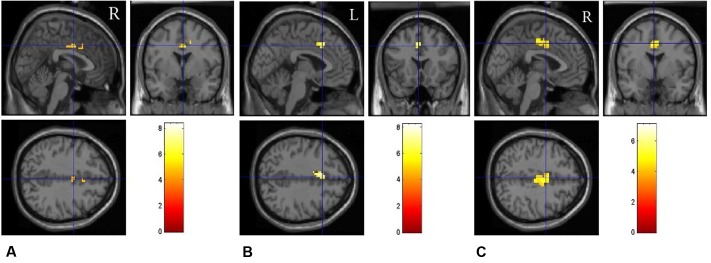
Brain regions involved in task switching in different emotional conditions. The fMRI results are shown separately for the neutral **(A)**, positive **(B)**, and negative **(C)** emotional conditions, respectively.

We first performed the contrast “*switch*” vs. “*repeat*” trials on fMRI data to explore the neural correlates underlying task switching. The main effect of Trial type (switch vs. repeat) resulted in large clusters of activation covering ACC for all the emotion states, *F*(1,17) = 28.38, *p* < 0.001, indicating higher BOLD responses in the switch trials than those in the repeat trials. We analyzed the contrast between “switch” trials and “repeat” trials for each emotional condition, and the results showed that there existed significant BOLD responses in the bilateral cingulate gyrus (BA 10) for the neutral emotion condition (**Figure 4A**), *t*(17) = 5.26, *p* < 0.01. For the positive emotion condition, the left ACC (BA 32) showed higher BOLD responses in the switch trials than those in the repeat trials (**Figure 4B**), *t*(17) = 4.39, *p* < 0.01. For the negative emotion condition, significant activation was observed in the bilateral ACC [*t*(17) = 5.21, *p* < 0.01], left putamen [*t*(17) = 4.11, *p* < 0.01], left medial globus pallidus [*t*(17) = 3.97, *p* < 0.05] and right frontal regions [*t*(17) = 4.13, *p* < 0.01] (**Figure 4C**). **Figure 4** and **Table 3** illustrate the cortical areas which were mainly activated during switch trials. **Table 3** shows all the significantly activated brain regions in the above contrasts in terms of peak coordinates, *t* scores, and extent of activation. All brain regions included in a single cluster of activation are listed within the table and clusters are ordered according to the significance of the peak voxel.

**Table 3:** Brain regions related to task switching (switch vs. repeat) under different emotional conditions.

Contrast condition	Brain regions	BA	Voxels	Cluster peak (mm)			*t*
Neutral-(switch vs. repeat)	R cingulate gyrus	10	56	9	11	30	5.26
Positive-(switch vs. repeat)	L cingulate gyrus	32	32	-4	16	33	4.39
Negative-(switch vs. repeat)	R cingulate gyrus	24	176	5	-4	42	5.21
	L cingulate gyrus	32	47	-15	-48	26	4.35
	L precuneus	7	37	-15	41	52	4.36
	L putamen		72	-28	-20	2	4.11
	L medial globus pallidus		38	-18	-9	0	3.97
	R frontal lobe		30	-21	-13	28	4.13


*The coordinates (x, y, z) are in the Talairach coordinates. BA, Brodmann’s areas; L, left; R, right. Displayed are the coordinates of the maximally activated voxel within a significant cluster.*

#### Influence of Emotional Valence upon Reducing Conflict for Switch Trials

**FIGURE 5:**
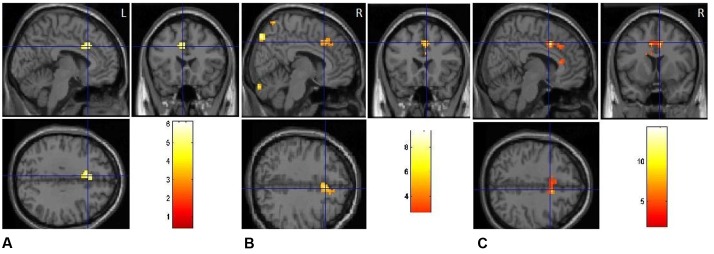
Emotional priming modulates response conflict in anterior cingulate. **(A)** Positive-switch trials vs. neutral-switch trials. **(B)** Negative-switch trials vs. neutral-switch trials. **(C)** Negative-switch trials vs. positive-switch trials.

**FIGURE 6:**
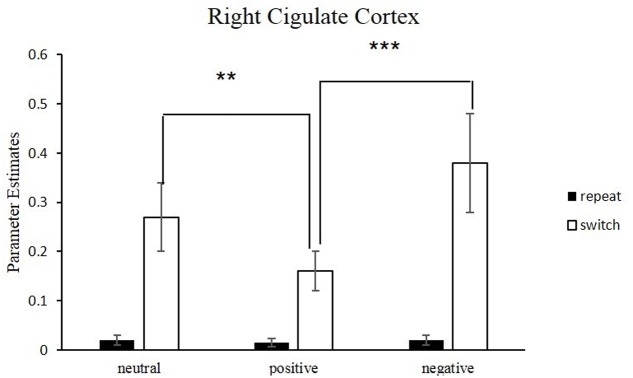
Neural correlates underlying switch vs. repeat conditions for different emotional conditions. Error bars indicate the standard deviation. Conditions denoted by two asterisks indicate significant difference between them (^∗∗^*p* < 0.01; ^∗∗∗^*p* < 0.001).

**Figure 5** and **Table 4** depict the activation of ACC under different emotional states for switch trials. Right cingulate cortex was significantly involved in the neural interaction of Trial type and Emotion states, *F*(2,34) = 21.97, *p* < 0.001. This interaction (**Figure 6**) was due to less activation of right cingulate cortex in the positive condition than in the neutral condition for switch trials [*t*(17) = -4.26, *p* < 0.01] and was due to decreased activation in the positive condition relative to the negative condition during switch trials [*t*(17) = -6.20, *p* < 0.001].

**Table 4:** Brain regions under different emotional states for switch trials.

Contrast condition	Brain regions	BA	Voxels	Cluster peak (mm)			*t*
Switch-(neutral vs. positive)	L cingulate gyrus	32	38	-6	22	29	4.98
Switch-(negative vs. neutral)	R parietal lobe	7	144	9	-76	47	4.77
	R occipital Lobe	17	101	6	85	-14	4.64
	R cingulate gyrus	32	82	6	23	35	4.60
	R anterior cingulate	33	84	6	21	21	5.45
	R medial frontal gyrus	12	80	9	37	31	3.87
	L thalamus		29	-18	20	9	4.06
	L lateral globus pallidus		23	-24	-17	1	3.68
	L inferior temporal gyrus	20	44	-51	-70	1	4.39
	L middle occipital gyrus	19	30	-54	-64	-4	4.28
Switch-(negative vs. positive)	R cingulate gyrus	32	137	9	13	35	6.20
	L cingulate gyrus	32	137	-6	11	35	6.11
	L corpus callosum		98	-12	13	22	4.56
	R anterior cingulate	33	64	12	30	10	4.17


*The coordinate (x, y, z) are in the Talairach coordinates. BA, Brodmann’s areas; L, left; R, right. Displayed are the coordinates of the maximally activated voxel within a significant cluster.*

For switch trials, compared to neutral condition, the BOLD response in left ACC (BA32) decreased significantly in the positive emotional condition, with the peak voxel lying in the left cingulate gyrus (**Figure 5A**), *t*(17) = 4.98, *p* < 0.01. By contrast, significant activation was observed in the bilateral anterior cingulate gyrus (BA32) by comparing negative-switch condition with positive-switch condition (**Figure 5C**), *t*(17) = 6.09, *p* < 0.001, indicating that larger activation in ACC for negative-switch trials than for positive-switch trials. Moreover, relative to neural-switch emotion condition, significant activation of dorsal ACC [*t*(17) = 5.43, *p* < 0.01] was observed in the negative-switch emotion condition (**Figure 5B**). Since the activation of dorsal ACC was generally involved in competition monitoring and conflict resolving, these results revealed that positive emotion state could reduce the activation of ACC related to conflict and facilitate cognitive flexibility, whereas negative emotional state inhibited cognitive flexibility.

#### Influence of Emotional Valence upon Reducing Conflict for Repeat Trials

We compared the differences of activation under three emotional conditions for repeat trials. No significant difference was observed for the activation of ACC in repeat trials across three conditions *F*(2, 34) = 0.49, *p* > 0.05.

## Discussion

The present study examined whether positive emotion could promote cognitive flexibility and the underlying brain mechanisms. Our results provided the evidence that cognitive flexibility was modulated by emotional states. Behaviorally, switch cost was reduced under positive emotion compared with negative or neutral emotion. Accordingly, the activation in dorsal ACC reduced significantly in switch trials under positive emotion state. By contrast, the activation of dorsal ACC increased significantly under negative emotion condition for switch trials.

### Emotion-Related Brain Activity

In the present study, the brain activity in occipital regions increased when viewing emotional pictures. Firstly, we found activation in medial orbitofrontal cortex (OFC) for positive emotion, which was consistent with prior findings with IAPS pictures (Kensinger and Schacter, 2006; Vink et al., 2014), indicating that this region was involved in processing of pleasant or rewarding stimuli. In addition, we found activations in the frontal cortex, including medial prefrontal cortex, superior frontal cortex, middle frontal cortex as well as the occipital cortex. For example, the activation of medial frontal cortex increased when participants watched films about a love story (Matsunaga et al., 2009). Secondly, encoding of negative IAPS pictures produced greater brain activation in middle frontal gyrus, middle occipital gyrus, right amygdala, left parahippocampal gyrus, fusiform gyrus and putamen than for the neutral pictures. These results are in line with recent functional imaging studies of human amygdala, in which activation of amygdala reflects the integration of perceptual information and emotional associations of the stimuli (Klinge et al., 2010). The amygdala plays a central role in assessing emotional salience, processing negative facial expressions, and particularly generation of aversive affective state (Costafreda et al., 2008). In addition, we found that negative emotional stimuli also activated the parahippocampus, which was involved in establishing and maintaining memory traces (Squire et al., 2007). The activation of parahippocampal gyrus indicates that memory traces must be activated upon the presentation of emotionally relevant stimuli, so that these stimuli can be recognized, related to past and present contextual experiences, and ultimately induce emotional state (Colibazzi et al., 2010). The concurrent activation of the amygdala, and parahippocampus further supports that the amygdala-hippocampal network is involved in the consolidation of emotional memories (Ritchey et al., 2008; Chan et al., 2014).

### Conflict-Related Brain Activity

In all emotion conditions, we observed that behavior flexibility was associated with brain activation of bilateral anterior cingulate gyrus and bilateral rostral anterior cingulate cortex in a task-switching paradigm (Waegeman et al., 2014). It appears that conflict monitoring and response conflict may play an important role in the task-switching paradigm (Hakun and Ravizza, 2012). The current study showed that the dACC activity increased in switch trials relative to repeat trials. One explanation was that dACC activity was specifically engaged in processing conflict between competing stimuli or responses. A series of fMRI studies of cognitive flexibility consistently report activation of dorsal anterior cingulate cortex (BA24, 32) (Swainson et al., 2003; Maier and Di Pellegrino, 2012), suggesting that these cortical regions are involved in switching task. The dorsal ACC (BA 24/32) is involved in cognitive flexibility associated with monitoring conflict, detecting error, and changing current inappropriate behavior. Therefore, we replicated the previous findings that ACC was involved in response competition, cognitive conflict and cognitive flexibility (Clayson and Larson, 2012; Brown, 2013).

### Positive Emotion Facilitates Cognitive Flexibility

In this study, behavioral data showed that significant switch cost was observed only under neutral and negative conditions, but not under positive condition, indicating that positive emotion reduced response conflict. Consistent with behavioral results, our fMRI results showed that activation of ACC during task switch reduced under positive emotional state, indicating that positive emotion could facilitate cognitive flexibility. Some researchers identified that greater activation of ACC was associated with greater conflict. Thus, the current research extends previous findings on the role of the ACC in monitoring response conflict. When novel task is executed, specifically, necessitates cognitive flexibility, the decreased BOLD response in ACC may reflect a type of conflict adaptation.

In this study, we found that the activation of ACC decreased in positive emotion conditions, but increased in negative emotions when participants performed a switch task, and the modulation of dACC activity was associated with RTs. The fact suggests that positive emotions and negative emotions affect response conflict differently both at the neural and behavioral levels. One interpretation of these findings is that positive emotions enhance cognitive control and reduce sensitivity to response conflict. Such a “de-sensitization” to response conflict might arise naturally as positive emotions activated reward-related brain regions.

The results of the current study are in line with the neuropsychological theory of positive emotion that positive emotion is associated with enhanced brain dopamine levels (Ashby et al., 1999). Since dorsal anterior cingulate is involved in the selection of cognitive perspective and set-switching (Posner and Raichle, 1994), the increased dopamine projection into the anterior cingulate facilitates executive attention and the selection of cognitive perspective. Due to increased dopamine release in the anterior cingulate, positive emotion is likely to improve cognitive flexibility measured by task-switching. The results of the current study are also in consistent with the conflict-monitoring theory (Botvinick et al., 2004; Botvinick, 2007). According to the conflict-monitoring theory, the activation of ACC is the greatest for trials with the longest RTs. Moreover, more conflict could induce larger activity in ACC following switch trials than repeat trials. The present findings indicate that positive emotion enhances the ability of cognitive control, and reduces the activation of brain areas related to conflict. However, further research is needed to understand exact association between decreased activation of dACC and positive emotion.

### Negative Emotion Increased Conflict-Related Activity

In the present study, the activation of ACC increased during negative emotional state, which was in consistent with early study that the activity of ACC increased during negative emotional states (Ichikawa et al., 2011). In their study, they found that the activity of dACC increased after errors. Moreover, the dACC showed higher activation in the increased negative emotion condition and lower activation in the decreased negative emotion condition, as compared to the neutral condition in their study. Their results could be explained that negative-relevant stimulus partially drained attentional resources that would have otherwise been allocated to the target (Meinhardt and Pekrun, 2003). Therefore, participants need more attentional control to accomplish experimental task under negative emotional state. Thus, the current results are partially consistent with these interpretations that participants need more cognitive resources to process negative emotion and show more errors when performing cognitive task, eliciting higher activation in dACC which is involved in cognitive flexibility.

In summary, we examined the effects of different emotional states on brain activity associated with cognitive flexibility using a task-switching paradigm. The results suggested emotional states modulated activities of ACC in response to switch trials. Positive emotional state reduced switch cost and reduced the activation of dACC involved in task switching, whereas negative emotions state increased the activation of dACC. Since emotional stimuli used in the present study were mild arousal stimuli, our results showed that the modulation of emotion on the cognitive flexibility was driven by valence. Future studies could elucidate the separate contributions of emotional arousal and valence to cognitive flexibility.

## Ethics Statement

All subjects gave written informed consent in accordance with the Declaration of Helsinki. The protocol was approved by the Ethics Committee of Capital Normal University.

## Author Contributions

Concept and design of study: YW and ZY. Data acquisition, analysis and interpretation: YW and JC. Drafting the work or revising it critically for important intellectual content: YW and ZY. Final approval of the version to be published: YW, ZY, and JC. Agreement to be accountable for all aspects of the work in ensuring that questions related to the accuracy or integrity of any part of the work are appropriately investigated and resolved: YW

## Conflict of Interest Statement

The authors declare that the research was conducted in the absence of any commercial or financial relationships that could be construed as a potential conflict of interest.

## Footnotes

1 http://www.fil.ion.ucl.ac.uk/spm/software/
